# Re-evaluation of hypoplastic left heart syndrome from a developmental and morphological perspective

**DOI:** 10.1186/s13023-017-0683-4

**Published:** 2017-08-10

**Authors:** A. Crucean, A. Alqahtani, D. J. Barron, W. J. Brawn, R. V. Richardson, J. O’Sullivan, R. H. Anderson, D. J. Henderson, B. Chaudhry

**Affiliations:** 1 Department of Cardiac Surgery, Birmingham Children’s Hospital, Birmingham, B4 6NH UK; 2 Cardiovascular Research Centre, Institute of Genetic Medicine, Newcastle University, Central Parkway, Newcastle upon Tyne, NE1 3BZ UK; 3 Department of Congenital Cardiology, Newcastle upon Tyne Hospitals NHS Foundation Trust, Newcastle upon Tyne, NE7 7DN UK

**Keywords:** Hypoplastic left heart, Congenital heart defects, Anatomy, Developmental, Lineage tracing, Mouse models, Aetiology

## Abstract

**Background:**

Hypoplastic left heart syndrome (HLHS) covers a spectrum of rare congenital anomalies characterised by a non-apex forming left ventricle and stenosis/atresia of the mitral and aortic valves. Despite many studies, the causes of HLHS remain unclear and there are conflicting views regarding the role of flow, valvar or myocardial abnormalities in its pathogenesis, all of which were proposed prior to the description of the second heart field. Our aim was to re-evaluate the patterns of malformation in HLHS in relation to recognised cardiac progenitor populations, with a view to providing aetiologically useful sub-groupings for genomic studies.

**Results:**

We examined 78 hearts previously classified as HLHS, with subtypes based on valve patency, and re-categorised them based on their objective ventricular phenotype. Three distinct subgroups could be identified: slit-like left ventricle (24%); miniaturised left ventricle (6%); and thickened left ventricle with endocardial fibroelastosis (EFE; 70%). Slit-like ventricles were always found in combination with aortic atresia and mitral atresia. Miniaturised left ventricles all had normally formed, though smaller aortic and mitral valves. The remaining group were found to have a range of aortic valve malformations associated with thickened left ventricular walls despite being described as either atresia or stenosis. The degree of myocardial thickening was not correlated to the degree of valvar stenosis. Lineage tracing in mice to investigate the progenitor populations that form the parts of the heart disrupted by HLHS showed that whereas *Nkx2–5-Cre* labelled myocardial and endothelial cells within the left and right ventricles, *Mef2c-AHF-Cre*, which labels second heart field-derived cells only, was largely restricted to the endocardium and myocardium of the right ventricle. However, like *Nkx2–5-Cre*, *Mef2c-AHF-Cre* lineage cells made a significant contribution to the aortic and mitral valves. In contrast, *Wnt1-Cre* made a major contribution only to the aortic valve. This suggests that discrete cardiac progenitors might be responsible for the patterns of defects observed in the distinct ventricular sub-groups.

**Conclusions:**

Only the slit-like ventricle grouping was found to map to the current nomenclature: the combination of mitral atresia with aortic atresia. It appears that slit-like and miniature ventricles also form discrete sub-groups. Thus, reclassification of HLHS into subgroups based on ventricular phenotype, might be useful in genetic and developmental studies in investigating the aetiology of this severe malformation syndrome.

## Background

Hypoplastic left heart syndrome (HLHS) is the term applied by Noonan and Nadas [1] to a range of rare congenital malformations initially described by Lev [2], which are characterised by a non-apex forming left ventricle and a narrowed ascending aorta. Additional key features of mitral and aortic valve atresia or stenosis provide the basis of a descriptive clinical classification. Exclusion criteria include double outlet right ventricle, atrioventricular septal defects and large ventricular septal defects [3]. Despite improved survival since the introduction of staged uni-ventricular surgical repair by Norwood [4] the aetiology of HLHS still remains unclear. Non-genetic causes, such as solvent exposure [5], viral infection [6] and disturbances of intra-cardiac blood flow [7] have been suggested. Based on its familial recurrence, a genetic component has been recognised [8–10] but genome-wide association studies (GWAS) have not yielded novel candidate genes [11]. Similarly, a small number of well-known genes involved in cardiogenesis, such as *GJA1* [12, 13], *HAND1* [14, 15], *NOTCH1* [16], *NKX2–5* [15, 17] and *GATA4* [15] have been suggested but have not been confirmed as being significant causes of HLHS.

In contrast to the limited progress in finding the causes of HLHS, there has been a revolution in our understanding of cardiac morphogenesis since the description of the second heart field (SHF) in 2001. The previous theory proposing segmental patterning within the primitive heart tube was an attractive one, not least because it apparently explained many heart defects including HLHS. We now know it to be incorrect. Instead, it is recognised that a single atrial and ventricular chamber are initially formed from the first heart field (FHF), but that consequent addition of cells from the surrounding SHF produce parts of the atria, the right ventricle and the outflow tract (reviewed in [18]). Studies using knockout and transgenic mice have indicated the importance of other multipotent progenitor populations in forming the heart including: neural crest cells; mesenchymal cells formed by endothelial to mesenchymal transformation (EndoMT); smooth muscle cells and fibroblasts derived by epithelial to mesenchymal transformation (EMT) from the epicardium (reviewed in [19]). It is an appropriate moment, therefore, to re-evaluate HLHS from a developmental perspective.

In this study, we take a novel approach to understanding HLHS, initially by reviewing the intra-cardiac morphology of hearts appropriately coded as HLHS, looking specifically for patterns of abnormalities that would correlate with disturbance of known developmental processes. Then, using genetic lineage tracing with *Cre-lox* mice, we ask if the features of HLHS subtypes can be adequately explained by abnormalities attributable to specific progenitor populations. We demonstrate three discrete ventricular phenotypes, but note that these are associated with a broad range of abnormalities affecting the aortic and mitral valves suggesting that HLHS may be the result of distinct developmental sequences. In some situations, valvar abnormalities might be the initiating abnormality, whilst in others the primary issue most likely lies in abnormal endocardial or myocardial growth or signalling. This study indicates how the heterogeneity of phenotype in HLHS can be grouped in studies aiming to explain the pathogenesis of HLHS.

## Methods

### Archival hearts

The congenitally malformed heart archive at Birmingham Children’s Hospital contains approximately 2000 specimens collected since 1939. Permission to study the collection was granted by the R&D department at Birmingham Children’s Hospital NHS Foundation Trust and the Custodian Committee of the collection. Seventy-eight un-operated hearts, in good condition and fulfilling the accepted criteria for HLHS [3], were identified. The hearts had been already prosected, fixed and stored in formalin; it was not permissible to undertake further prosection. Demographic data such as age and sex were not available. Measurements of the hearts were made using Vernier callipers (Fisher Scientific), with photography carried out using a digital single lens reflex camera with macroscopic lens (Nikon 3100, Nikon, Japan) or stereomicroscope (MZ16, Leica, Germany).

### Animals

*Mef2c-AHF-Cre*, *Wnt1-Cre*, *Nkx2–5-Cre*, and *Tie2-Cre* [20–23] mice, together with *ROSAeYFP* and *mTmG* reporter lines were used in these experiments. Specificity of expression of these *Cre* drivers in our hands has been confirmed in previous studies [24, 25]. The studies were approved by the Newcastle University Animal Welfare and Ethical Review Board and conformed to the Animals (Scientific) Act 1986 (UK) and Directive 2010/63/EU of the European Parliament.

### Staining and immunohistochemistry

Mouse embryos were collected at embryonic (E) days 10.5 and E15.5. Immunohistochemistry was carried out as described previously [24, 26]. Alexa 488 or Alexa 594 (Life Technologies) secondary antibodies were used for immunofluorescence. Images were acquired using an Axioimager II and Apotome II (Zeiss, Jena, DE).

### Statistics

Comparisons were made using ANOVA (SPSS 14.0, IBM) assuming non-parametric distributions. Post Hoc testing with Dunn’s test allowed for unequal group size.

## Results

**Fig. 1:**
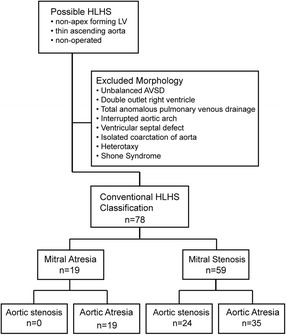
Conventional classification of HLHS hearts. Selection of 78 hearts with conventional HLHS. Other cardiac malformations associated with a small left ventricle were excluded. No hearts with mitral atresia and aortic stenosis were included as these hearts have an associated ventricular septal defect

Based on macroscopic examination and probe patency, we sub-classified 78 hearts with a previous diagnosis of HLHS (Fig. 1), focussing on the ventricular phenotype. All hearts had normal arrangements and connections between the atria, ventricles and great vessels. The right ventricles and connected valves were normal and in all cases a patent foramen ovale guarded by a patulous, probe patent, flap valve was found. It was not possible to reliably identify the attachment of the pulmonary veins or the presence of persisting left-sided superior caval veins due to previous dissection.

### Three distinct subgroups of left ventricular phenotype

**Fig. 2:**
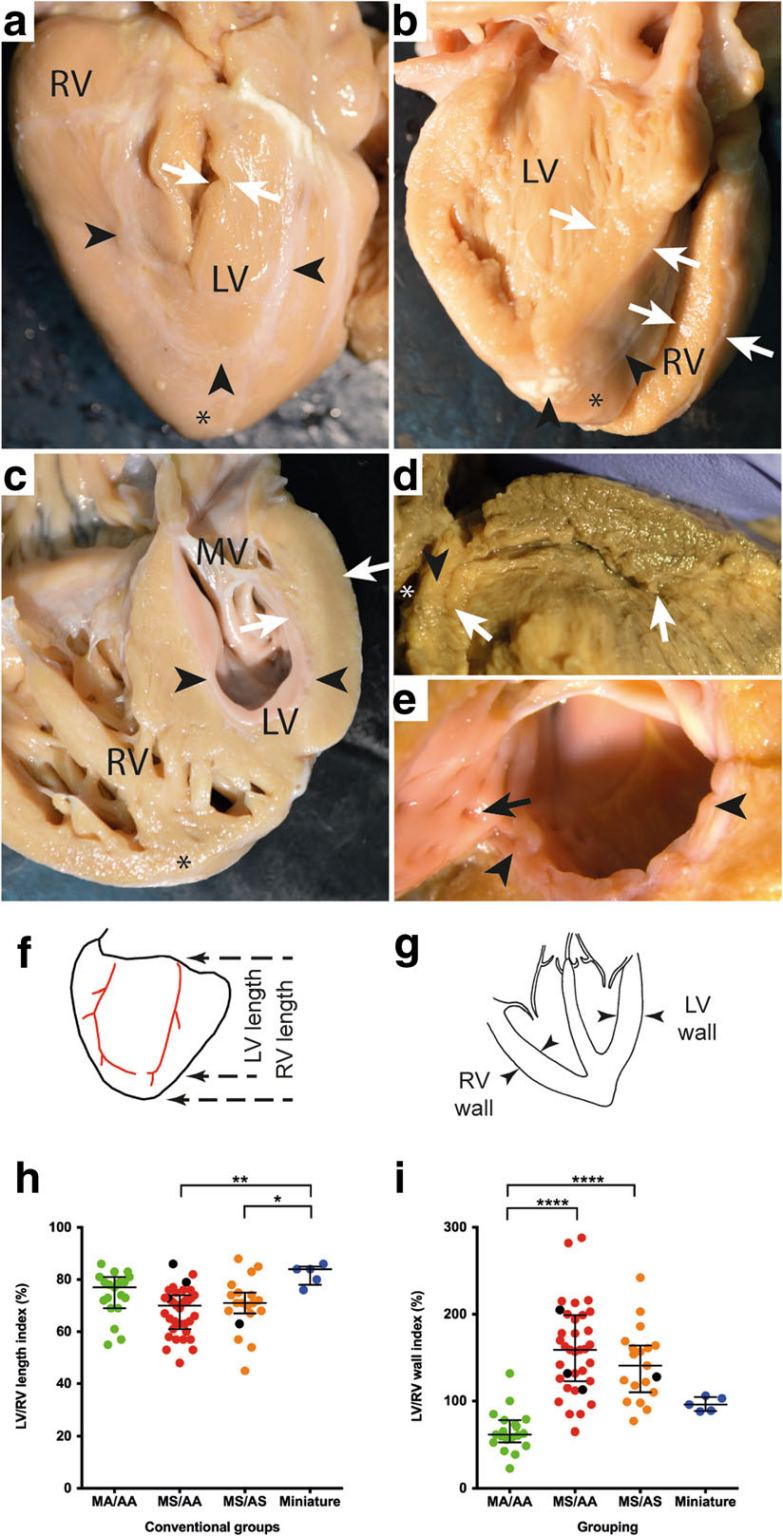
Ventricular phenotypes in HLHS. **a** posterior view of heart with slit-like ventricle. The apex (*) is formed by the right ventricle (RV) and inter-ventricular coronary arteries (*arrowheads*) indicate the extent of the left ventricular cavity (LV), which has been opened with a longitudinal incision. The free wall is thin (*arrows*) and there is no endocardial fibroelastosis (EFE) within the cavity. **b** Miniaturised left ventricle. Although the apex (*) is formed by the right ventricle (RV) the left ventricular cavity is large as is the mitral valve. The left ventricular free wall is of similar thickness to the right ventricular free wall (*arrows*) and there is no EFE. **c** Heart with thick-walled (*arrows*) left ventricle. The right ventricle, containing coarse trabeculations, wraps around this to form the apex (*). There is prominent EFE (arrowheads) which also involves the mitral valve (MV). **d** Heart with slit-like ventricle in which the thin but long LV cavity (arrows) has been exposed by cutting longitudinally through the entire heart. There is no connection (*arrowhead*) with the left atrium (*). **e** EFE (*arrowheads*) with investment of left ventricular trabeculations (*arrow*). **f** Measurements of left ventricular length in relation to right ventricular length and **g** left ventricular free wall thickness in relation to right free wall thickness. **h** The ratio of LV/RV lengths of hearts with mitral atresia/aortic atresia (MA/AA), mitral stenosis/aortic atresia (MS/AA) and mitral stenosis/aortic stenosis (MS/AS) were the same (median and interquartile range). However, the miniature ventricles had longer ventricular cavities. **i** The LV/RV wall ratio in slit-like ventricles was reduced in comparison with other groups. Hearts with mitral stenosis/aortic atresia and mitral stenosis/mitral stenosis demonstrated LV wall thickening of similar extent. The LV and RV wall thicknesses were equal in hearts with miniaturised ventricles. *p < 0.05, **p < 0.01, ****p < 0.001

Examination of the left ventricles in HLHS specimens revealed three different appearances (Fig. 2). A distinctive subset, that we labelled as “slit-like ventricle”, was seen in 19/78 (24%) hearts and was characterised by a flattened left ventricle contained within the left-posterior aspect of the ventricular mass. The presence of the ventricle was suggested by delimiting coronary vessels and in all 19 hearts, a cross sectional incision revealed a thin parietal left ventricular wall and a slit-like cavity. All of these hearts had mitral atresia and aortic atresia (MA/AA, Fig. 2a, d, h, i). In contrast, the left ventricles in 5/78 (6%) hearts were almost normal in size and mural thickness, but did not form the cardiac apex (Fig. 2b, h, i). Although smaller than their right-sided counterparts, the aortic and mitral valves were anatomically normal. The remaining 54 hearts (70%) had a small left ventricular cavity, with the right ventricle forming the apex to the heart, but their left ventricular walls were markedly thickened (Fig. 2c, h, i). Of this group with “thickened parietal walls” 35/54 of these hearts were classified as examples of mitral stenosis/aortic stenosis (MS/AS), whilst 19/54 were considered as exhibiting mitral stenosis and aortic atresia (MS/AA; Fig. 2). Endocardial fibroelastosis (EFE), recognisable as a firm and pitted layer on the left ventricular endothelial surface, was present in 50/54 hearts with a thickened left ventricular parietal wall (Fig. 2e), but was not found in any of the hearts with slit-like or miniaturised left ventricles. Notably, although length of the left ventricle relative to the right ventricle was not a useful measure (Fig. 2h), the thickness of the left ventricular parietal wall (Fig. 2i) was a more pronounced method of distinguishing between the subgroups, particularly in combination with the presence or absence of EFE.

### Aortic and mitral valve phenotypes overlap between the three ventricular subgroups

**Fig. 3:**
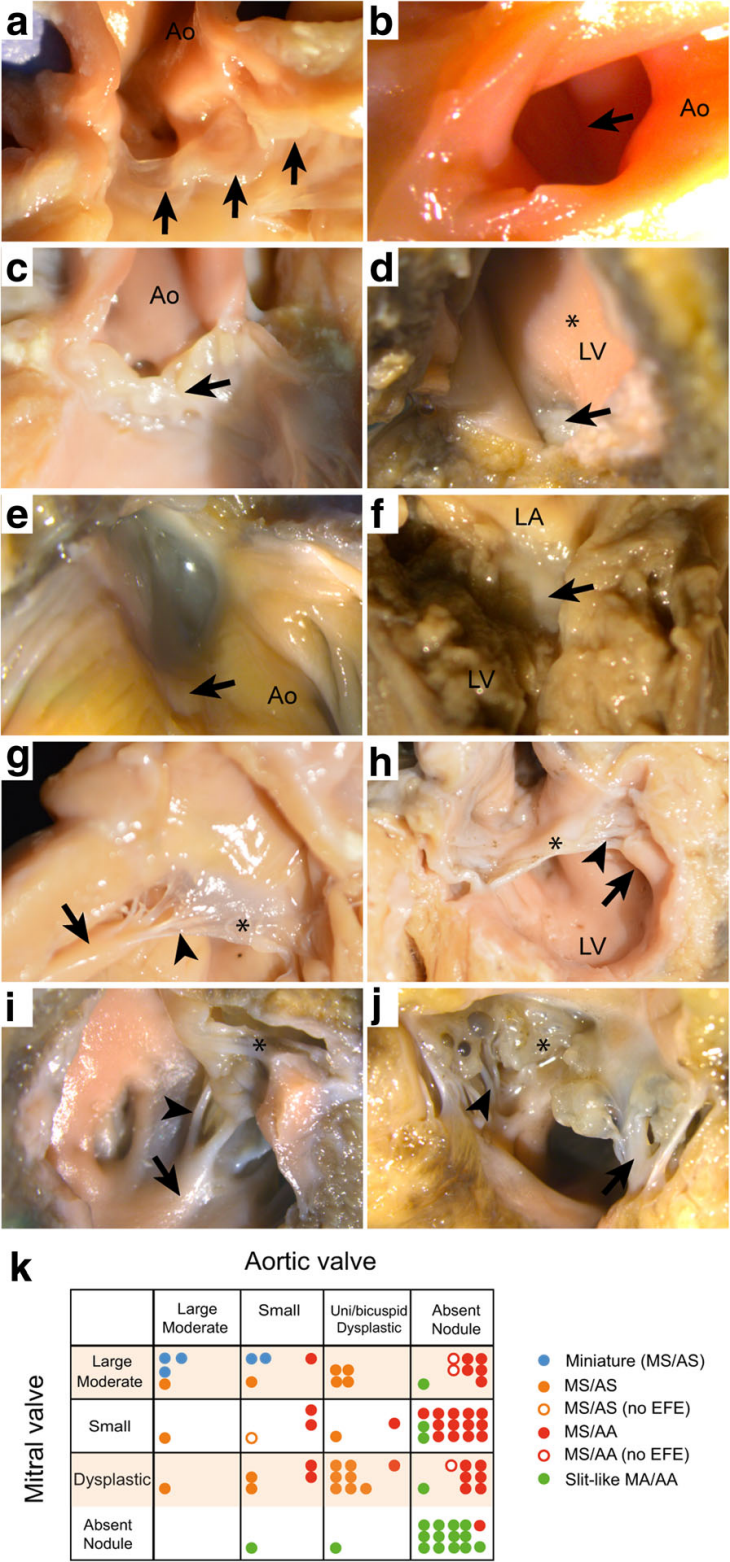
Appearances of mitral and aortic valves. Aortic (**a**-**e**) and mitral (**f**-**j**) valves in HLHS. A large well-formed aortic valve with thin leaflets in miniature ventricle. Arrows indicates three leaflets of aortic valve. **b** Small bicuspid aortic valve viewed from opened aorta (Ao) in MS/AA. Fused but apparently bi-foliate (*arrow*) aortic valve. **c** Aortic valve in AS/MS with uni-commisural valve and nodular dysplastic appearance (*arrow*). **d** Small nodule of valve tissue (*arrow*) in MS/AA ventricle seen from ventricular aspect with prominent EFE (*). **e** same valve from opened aorta. **f** nodule of white tissue indicating mitral atresia in slit like ventricle. **g** Mitral valve in heart with miniaturised ventricle. The mitral valve leaflet is thin (*) with well separated fine chordae (*arrowhead*) and a prominent papillary muscle (*arrow*); there is no EFE. **h** mitral valve with thickened leaflets (*), Short thick, but separate chordae (*arrowhead*) and small papillary muscles (*arrow*); marked EFE on LV surface. **i** Small stenotic mitral valve from MS/AA heart. leaflet (*), chordae (*arrowhead*) and papillary muscles (*arrow*) are coated with EFE. **j** Dysplastic mitral valve with nodular leaflets (*), sort chordae (*arrowhead*) and EFE coating small papillary muscle (*arrow*). **k** Correlation between mitral valve and aortic valve appearances

**Fig. 4:**
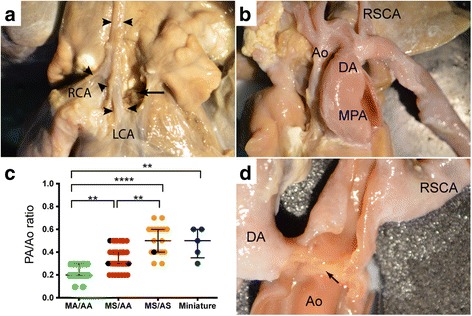
Reduced diameter of ascending aorta and ventricular phenotype in HLHS **a** Atretic ascending aorta (Ao) of similar diameter to left and right coronary arteries (LCA, RCA) in heat with MA/AA. The aortic root has been dissected but no aortic valve tissue is visible. **b** Larger calibre ascending aorta in MA/AS, with subclavian artery (RSCA) arising opposite ductus arteriosus (DA). **c** Diameter of ascending aorta in HLHS subgroups. There is an increase in the calibre of the ascending aortic segment in comparison to the pulmonary artery between ventricular phenotypic groups. The MA/AA group have the thinnest aorta, the MS/AA larger and the largest are in the MS/AS and miniature ventricle groups. **p < 0.01,****p < 0.001 **d**. Example of spillage of ductal tissue into aorta (*arrow*)

We next examined the aortic and mitral valves in more detail, relating them with the ventricular phenotypes as described above. Of the 24 hearts with aortic stenosis, all five with miniaturised ventricles contained remarkably large and well-formed aortic valves with three thin leaflets (Fig. 3a). The remaining 19/24 hearts with aortic stenosis had thick left ventricular walls and contained small and/or abnormal aortic valves (Fig. 3b, c, k). There were 54/78 hearts with aortic atresia and 19/54 of these had slit-like left ventricles. In 16/19 of these hearts it was not possible to identify the aortic valve (arrow in Fig. 4a), although a small area of white fibrous tissue was seen at the anticipated site of the leaflets in 2/19. Small, imperforate bicuspid leaflets were seen in the last case. No aortic valvar tissue could be found in the 28/35 of the remaining aortic atresia hearts with thickened ventricular wall. In the remaining 7/35 there was either an area of fibrous tissue (Fig. 3d, e) or fused valve leaflets. It was not possible to distinguish unicuspid from bicuspid valves with certainty, but the sizes of the imperforate valve were proportionate to the size of the aortic root.

Although a range of abnormalities was seen in the mitral valves, there was a general relationship between the size of papillary muscles, length of tendinous chords and the degree of leaflet formation. The largest valves with well-formed thin leaflets, fine separate tendinous cords, and prominent papillary muscles, were found in the five hearts with miniaturised ventricles (MS/AS) (Fig. 3g, k). In 15/19 of the hearts with slit-like ventricles (MA/AA), the mitral valves were either absent or represented by a small nodule of white tissue (Fig. 3f). The remaining 4/19 slit-like ventricles contained identifiable mitral valve structures. In three cases, they were either small or dysplastic, although in one the valve was of moderate size, but with fused leaflets. The remaining 35 hearts with aortic atresia had thickened left ventricular walls. They exhibited a wide range of mitral valve appearances including moderate sized valves with cords but usually no papillary muscles; smaller valves with thin leaflets, small cords, and no papillary muscles; and thickened or nodular dysplastic valves (Fig. 3h-k). There was some correlation between aortic and mitral valve appearances. The hearts with miniaturised ventricles tended to contain the largest and relatively best-formed mitral and aortic valves (Fig. 3g, k), whereas the hearts with slit-like ventricles contained the most rudimentary of valvar appearances (Fig. 3f, k). In general, the malformation of the aortic valve was more severe than that of the accompanying mitral valve (Fig. 3k).

### The ascending aorta dimensions relate to flow through the aortic valve

The ascending aorta was narrow in comparison to the pulmonary trunk in all the hearts (Fig. 4). The ratio of the aorta:pulmonary trunk hemi-circumference (Ao:PT ratio) appeared to reflect the likelihood and magnitude of forward flow through this segment. Thus, the smallest dimensions were seen in hearts with MA/AA (Fig. 4a), with the collapsed left ventricular chamber indicative of absence of any appreciable forward-flow through the ascending aorta. This was followed by the group with MS/AA (Fig. 4b, c). In contrast, the hearts with miniaturised left ventricles and (mild) MS/AS had the largest aortic segments, in keeping with their large valves and relatively well-formed left ventricular chambers. The remaining hearts with thickened ventricles fell between these groups (Fig. 4b). Paraductal coarctation was also commonly seen in these hearts (Fig. 4d). However, it was not possible to accurately determine the frequency of this as in many cases the historical dissection of the aorta and ductus arteriosus had confused the positioning of these ductal spillages.

### Lineage contributions to the ventricles in the context of HLHS

To investigate the developmental mechanisms underpinning the miniaturised, slit-like, and thickened with EFE categories of HLHS described above, we used *Cre-lox* based lineage tracing in transgenic mice to determine the progenitor contributions and developmental processes common to the left-sided structures of the heart, focussing on stages prior to and following cardiac septation (embryonic day (E) 10.5 and E15.5 respectively).

**Fig. 5:**
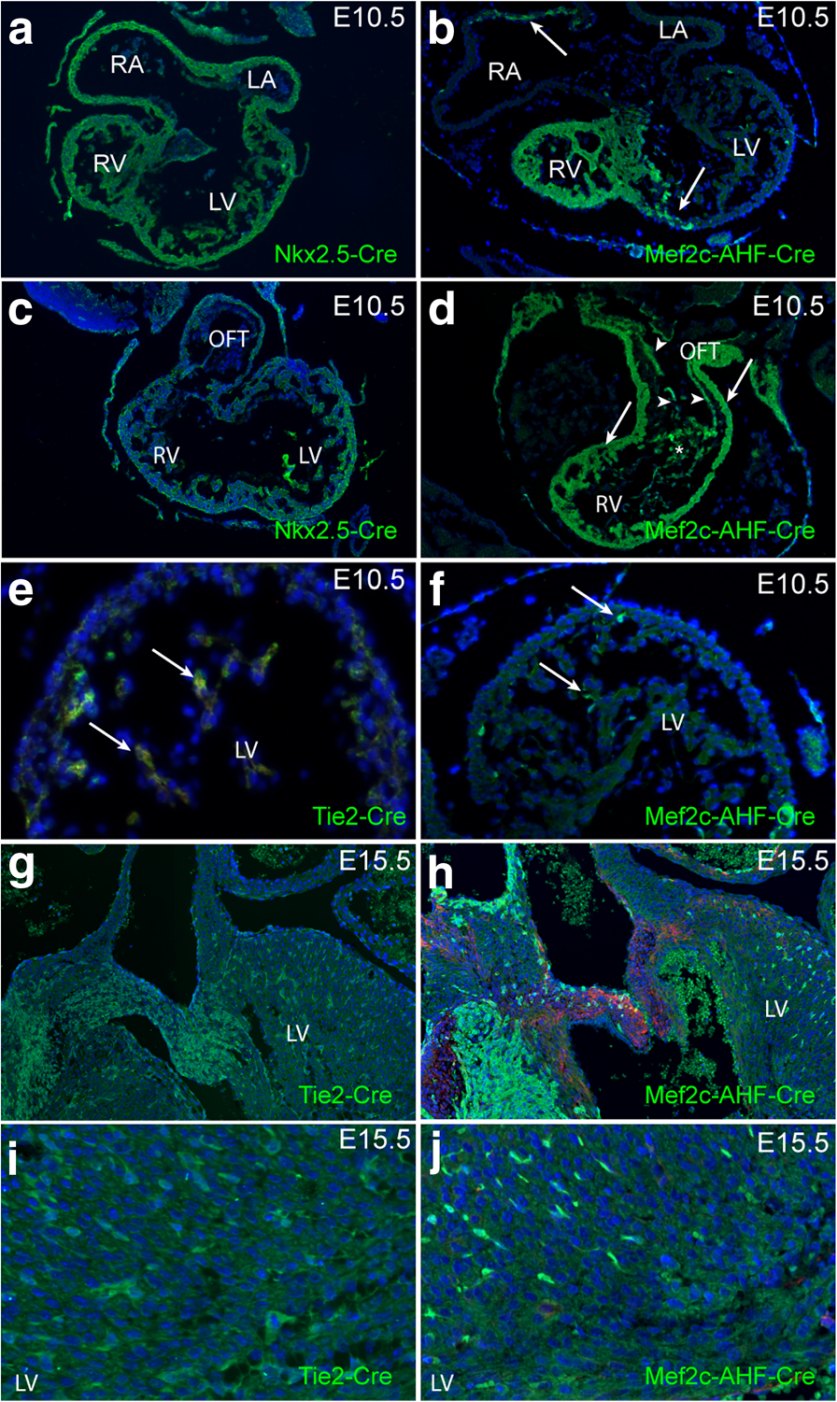
Lineage tracing of ventricular myocardium and endocardium. **a**, **c** The atrial, ventricular walls and outflow tract walls, including the myocardium and endocardium, are composed of cells from the *Nkx2–5-Cre* expressing lineage at E10.5 (*green*). **b** In contrast, *Mef2c-AHF-Cre*-expressing cells make only a minor contribution to the left ventricular myocardium and part of the right atrial roof (*arrows*). **d** *Mef2c-AHF-Cre*-expressing cells (*arrows*) form the right ventricle and outflow tract, and outflow endothelium (*arrowheads*), indicating origin from the SHF. **e** *Tie2Cre* is expressed in all of the cells in the left ventricular endocardium at E10.5, whereas *Mef2c-AHF-Cre* is expressed in only a small number (**f**). These patterns of staining for *Tie2-Cre* (**g**-**i**) and *Mef2c-AHF-Cr*e-expressing cells (**i**, **j**) are maintained at E15.5, with *Mef2c-AHF-Cre* labelling only a small portion of the endothelial cells in the left ventricle. *RA* right atrium, *LA* Left atrium, *RV* right ventricle, *LV* left ventricle, *OFT* outflow tract

Discrimination of FHF cells from SHF cells was performed by comparing the expression patterns of GFP reporter constructs in transgenic mice containing *Nkx2–5-Cre* that identifies all cardiomyocytes [22], with that generated using *Mef2c-AHF-Cre*, which specifically labels SHF-derived cardiomyocytes [20]. At E10.5, whilst *Nkx2–5-Cre* identified cardiomyocytes in the right and left atria and ventricles (Fig. 5a, c), *Mef2c-AHF-Cre* was restricted to the myocardium of the right ventricle and outflow tract, together with a region in the roof of the right atrium (Fig. 5b, d). The *Tie2-Cre* transgene labels all endocardium ([23]; Fig. 5e, g), whilst in contrast, *Mef2c-AHF-Cre* labels only SHF-derived endocardium (Fig. 5f, h). As with the myocardium, SHF-derived endothelial cells predominated in the right ventricle and outflow vessels (including the mature aorta), with very few *Mef2c-AHF-Cre*-labelled endocardial cells seen in the left ventricle (Fig. 5i, j; this was the only labelled patch of *Mef2c-AHF-Cre* labelled cells in the LV, found in the parietal wall, close to the mitral valve).

### Lineage contributions to the aortic and mitral valves in the context of HLHS

**Fig. 6:**
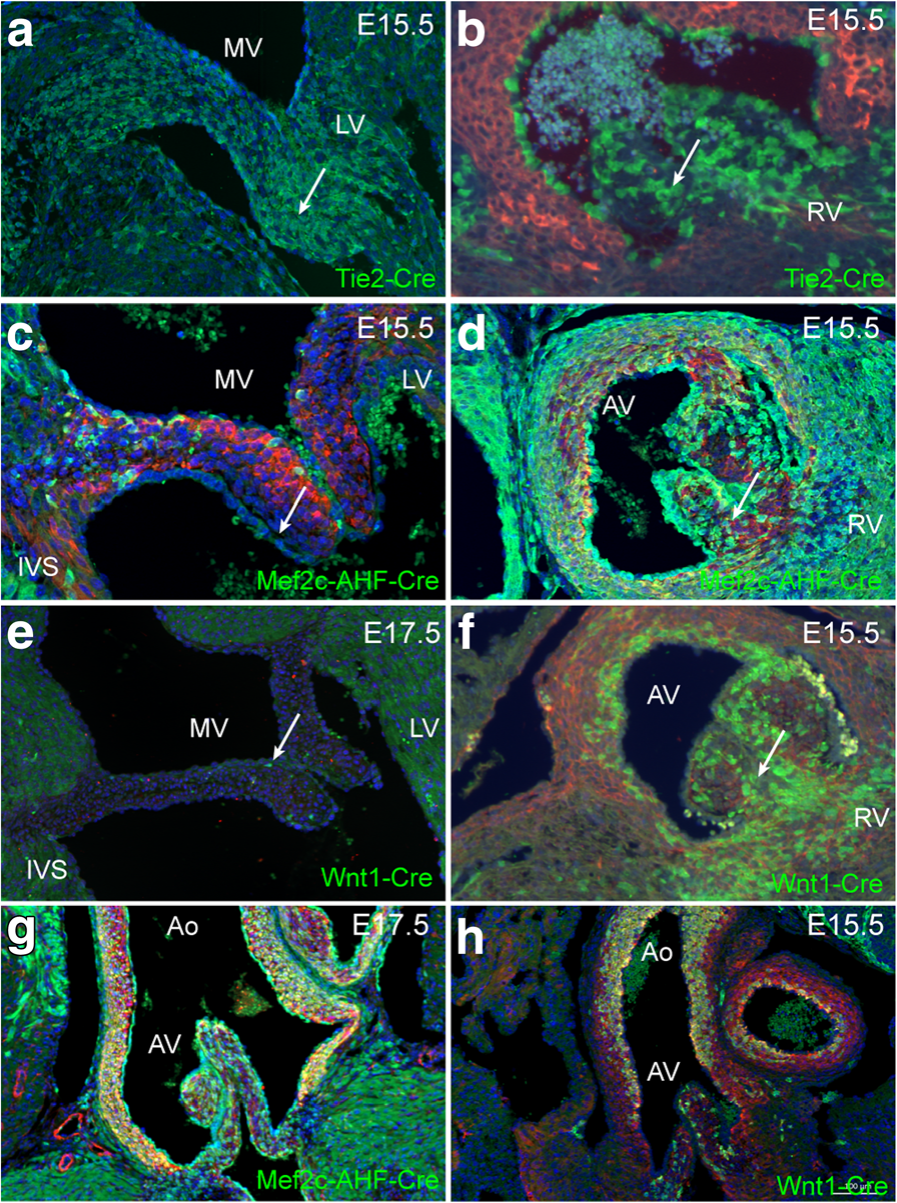
Lineage tracing of mitral and aortic valves and ascending aorta. *Tie2Cre*-labelled endothelial cells are abundant in the mitral (**a**) and aortic valves (**b**) at E15.5. *Mef2c-AHF-Cre*- labelled cells make a small contribution to the mitral valve (**c**) are also very abundant in the aortic valve (*arrow* in **d**). Although *Wnt1-Cre*-expressing neural crest cells are rare contributors to the mitral valve (*arrows* in **e**), they are abundant in the aortic valve (*arrow* in **f**). **g**, **h** *Mef2c-AHF-Cre* and *Wnt1-Cre* are both abundant in the ascending aorta at E15.5, although their patterns are distinct with more the *Mef2c-AHF-Cre*-expressing cells in the walls of the aortic root and greater numbers of *Wnt1-Cre*-expressing cells found more distally

*Mef2c-AHF-Cre* labelling showed that leaflets of both the mitral and aortic valves had an endothelial covering and contained interstitial cells of SHF-derived endothelial origin, that had undergone EndoMT (Fig. 6c, d; [23]). Cells within both the mitral and aortic valve were also identified by lineage tracing with *Mef2c-AHF-Cre*, indicating that the SHF makes a contribution to these endocardial-derived cells (Fig. 6c, d). Although *Wnt1-Cre*-labelled neural crest cells [21] were also abundant in the aortic valve and ascending aorta (Fig. 6e, f, h) there were none in the left ventricle. However, close examination of the mitral valve revealed a few cells derived from this lineage (Fig. 6e).

## Discussion

**Fig. 7:**
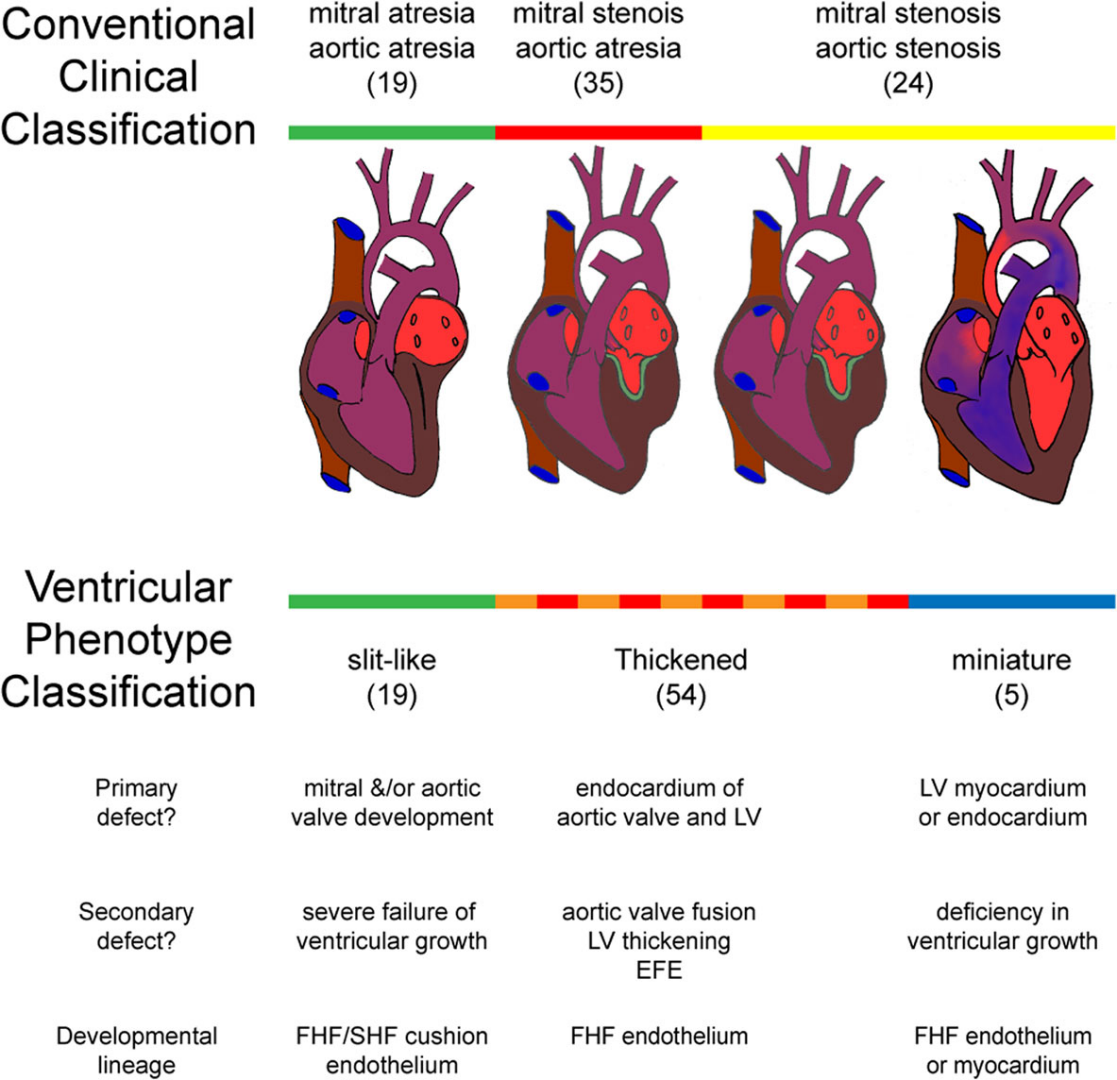
Comparison of classifications and relationship of defects and progenitor lineages. LV = left ventricle, EFE = endocardial fibroelastosis, FHF = first heart field and SHF = second heart field

Whilst heterogeneity of phenotype in HLHS is accepted [27] the possibility that HLHS is a collection of entirely separate entities has not, to the best of our knowledge, been mooted. In this study, which represents the spectrum of disease from the slit-like ventricle with MA/AA to the miniaturised left ventricle with mild MS/AS, we have shown how the conventional valve-based classification can be mapped to three distinctly different left ventricular phenotypes (Fig. 7). Furthermore, the parameters of wall thickness, LV cavity length and macroscopic evidence of EFE support this alternative view. This leads us to new hypotheses within the subgroups that are potentially testable and informative of aetiology. For example, it is possible that the slit-like ventricles arise due to a combined primary defect in the mitral and aortic valves. This then results in secondary failure of left ventricular development due to absent blood flow, combining a primary abnormality in valve development with the “no flow, no grow” hypothesis. Alternatively, the primary issue may lie in endothelial and/or myocardial signalling pathways promoting growth. This directs investigation towards understanding the nature of the fetal left ventricular myocardium in HLHS, both in slit-like ventricles and in those with thickened parietal walls, at early stages prior to secondary changes. Limited histological analyses have shown myofibrillar disarray in the thickened ventricular walls [28], but other studies have suggested common senescent changes [29]. Such early fetal samples, taken before secondary changes have become established are rare, but do occur. The timing of developmental aberrations also appears to be important in HLHS. Completion of ventricular septation prior to development of the valvar abnormalities may be an essential feature in the formation of the collapsed, slit-like ventricle in MA/AA [7], rather than leading to isolated mitral atresia with ventricular septal defect and maintained ventricular cavity volume. Ventricular septation is an early event, taking place in the 7th week of human embryonic development, thus placing the pathological mechanism underlying slit-like ventricle within a limited time window. In contrast, pre-natal diagnosis indicates that the thickened and miniaturised ventricular phenotypes can appear at different stages during the second and third trimester of pregnancy. These hearts exhibited a wide variety of aortic root and valve appearances. Some aortic valves were remarkably normal but within small roots, whereas others demonstrated markedly abnormal leaflets. This latter group may relate more strongly to familial aortic valve disease [9]. The hearts with miniaturised left ventricles, examples of the hypoplastic left heart complex [30], with only mildly-affected valve structures again form a phenotypically distinct group, The phenotype in this case is more in keeping with a generalised subtle limitation of growth of the left side of the heart, which could be due to endothelial sensing, communication or myocardial response. Thus, at this time, it appears that the slit-like ventricle, which fully maps to MA/AA, represents the most homogeneous group and therefore most suitable for initial genomic analyses. If genetic findings in this group can then be confirmed or otherwise in the other phenotypes this will be important in understanding pathogenesis and refining classification. It should be noted that all of the hearts examined here had probe-patent unrestricted intra-atrial communication and normal right-sided structures. This may be because the anatomical collection used in this study is biased to configurations that allowed initial postnatal survival. With this caveat, it is possible that other distinct sub-types might be present, that these additional features are variations based on the developmental sequence, or that they represent the effects of modifying mutations. In any event, identification of genetic pathways causing the principle ventricular HLHS phenotypes should clarify this. Similarly, we did not note macroscopic evidence of sinusoidal connections between the left ventricular cavity and coronary circulation [31], although this may have been apparent if histological examination was undertaken.

This study has emphasised the limitation of EFE to thickened ventricles, hearts conventionally described as severe MS/AS and MS/AA. Although sub-myocardial ischaemia has been proposed as a cause of EFE, analysis of coronary vasculature in HLHS has not supported this proposal [32, 33]. Recent studies have suggested that EFE results from excessive or ectopic endocardial to mesenchymal transformation [34, 35]. It is unclear whether this is therefore a specific element in the pathogenesis of this phenotype, in contrast to the miniaturised or slit-like ventricles, or represents a situation where there is reduced but present flow, signalling EFE formation as a secondary response. Our data suggests that the degree of ventricular wall thickening in the HLHS hearts did not correlate with the presence of aortic stenosis versus atresia, suggesting that these features are not directly linked. A correlation was seen between the diameter of the ascending aorta and aortic valve patency however, as has been described previously [36]. Although a significant role for flow in the growth of the ascending aorta has been suggested, and demonstrated in manipulations of embryonic circulation [7], the finding that the reduction in aortic dimensions in the miniaturised hearts was similar to that in hearts with thickened and extremely diminished left ventricular cavities was unexpected. The atretic ascending aortic segment develops from the aortic sac and shares SHF endothelial and smooth muscle contributions with the aortic root and valve. This raises the possibility that there may a developmental failure in growth of the ascending aorta in HLHS subgroups, that has not been distinguished from growth failure due to reduced flow.

To explore these concepts of embryonic origins further, we carried out a developmental analysis using *Cre-lox* based lineage tracing in mice. Mice are considered to be the best animal model for studying congenital heart disease on account of their close similarity in structure and developmental processes to human embryos. Indeed, where differences exist, they are associated with atrial and venous structures [37]. Recently, HLHS has been described in mice [38] although these mutant animals did not conform to the accepted human classifications [3], all presenting with double outlet right ventricle in combination with small left ventricles and hypoplastic aortas. *Cre-lox* technology has played a crucial role in uncovering the complex developmental roles of different cell lineages during heart development [18, 19]. The lines that we have utilised label all cardiomyocytes (*Nkx2–5-Cre*), second heart field-derived cells (*Mef2c-AHF-Cre*), endocardial cells (*Tie2-Cre*) and neural crest cells (*Wnt1-Cre*) [20–23] and were selected because they are reliable and reproducible and have been widely used, including by us, to investigate the roles of specific genes in these lineages (for example, [24, 25]). Although not analysed in our study, the epicardium is also an important cell population that contributes cells to the ventricular wall and atrioventricular valves, although no differences in distribution over the ventricular surfaces have been described [39]. Thus, there are a range of cell types that could be responsible for the phenotypes seen in HLHS. Our data supports the idea that some aspects of HLHS are likely to represent a developmental sequence rather than a co-existing series of primary defects [27]. Thus, in cases where there are isolated aortic valve abnormalities associated with thickened left ventricular parietal wall and EFE, an anomaly in the SHF or NCC cell types is extremely plausible, as these cell types make significant contributions to the forming aortic valve. Alternatively in other hearts in this same group it is possible that a primary myocardial (FHF) dysfunction could lead to myocardial thickening and secondary failure of aortic valve development consequent on reduced flow. In slit-like ventricles, the absence of normal mitral development, with or without normal aortic valve development, could lead to the collapsed left ventricle as these structures are adjacent in the early heart, abutting the inner curvature. The miniaturised ventricle phenotype supports the likelihood of a generalised anomaly in growth of the left side of the heart, involving both FHF-derived left ventricular endothelium and/or myocardium, as well as SHF-derived aortic endothelium. It seems unlikely that abnormalities in either the SHF or NCC could explain the phenotype in these hypoplastic left heart complex cases, and may explain why many succeed with biventricular repair [30].

## Conclusions

The new classification proposed in this study is not intended to replace conventional descriptions that are based on the valvar pathology. The conventional classification of HLHS has important clinical implications. For example, hearts with mitral stenosis and aortic atresia are associated with adverse clinical outcome due to sinusoidal connections between the coronary circulation and the left ventricular cavity [31, 40]. However, developmentally expressed genes can be restricted to first and second heart field lineages, as well as left and right-sided structures (reviewed in [19]). We suggest that the investigation of candidate HLHS genes suggested via genomic studies, should be studied within the context of progenitor contributions to specific affected structures. HLHS subgroups with a strong inheritance pattern may result from multigenic inheritance [9] or alternatively a simpler Mendelian inheritance complicated by incomplete penetrance and, as noted in previous studies, difficulties with heterogeneity of case selection [11].

## Abbreviations

AA: Aortic atresia
E: Embryonic day
EFE: Endocardial fibroelastosis
EMT: Epithelial to mesenchymal transformation
EndoMT: Endothelial to mesenchymal transformation
FHF: First heart field
GWAS: Genome wide association studies
HLHS: Hypoplastic left heart syndrome
MA: Mitral atresia
MS: Mitral stenosis
SHF: Second heart field
